# All-cause mortality in moderate and severe COVID-19 patients with myocardial injury receiving versus not receiving azvudine: a propensity score-matched analysis

**DOI:** 10.1097/CP9.0000000000000049

**Published:** 2023-05-31

**Authors:** Ru Chen, Yi Guo, Shan Deng, Jian Wang, Meng Gao, Hongli Han, Lin Wang, Hongwei Jiang, Kai Huang

**Affiliations:** 1 Department of Cardiology, Union Hospital, Tongji Medical College, Huazhong University of Science and Technology, Wuhan 430022, China.; 2 Clinic Center of Human Gene Research, Union Hospital, Tongji Medical College, Huazhong University of Science and Technology, Wuhan 430022, China.; 3 Department of Clinical Laboratory, Union Hospital, Tongji Medical College, Huazhong University of Science and Technology, Wuhan 430022, China.; 4 Liyuan Cardiovascular Center, Tongji Medical College, Huazhong University of Science and Technology, Wuhan 430022, China.; 5 Research Center for Tissue Engineering and Regenerative Medicine, Union Hospital, Tongji Medical College, Huazhong University of Science and Technology, Wuhan 430022, China.; 6 Department of Epidemiology and Biostatistics, Ministry of Education Key Laboratory of Environment and Health, School of Public Health, Tongji Medical College, Huazhong University of Science and Technology, Wuhan 430022, China.; 7 Hubei Key Laboratory of Metabolic Abnormalities and Vascular Aging, Huazhong University of Science and Technology, Wuhan 430022, China.; 8 Hubei clinical research center of metabolic and cardiovascular disease, Huazhong University of Science and Technology, Wuhan 430022, China.

**Keywords:** COVID-19, Myocardial injury, Azvudine, Mortality

## Abstract

Omicron is currently the dominant strain of severe acute respiratory syndrome coronavirus 2, but little is known about the characteristics and management of omicron related myocardial injury, particularly the potential benefit of the antiviral agent azvudine.

**Methods:**

Patients with confirmed and suspected coronavirus disease 2019 (COVID-19) admitted to Wuhan Union Hospital from December 7, 2022, to December 30, 2022, were included in this study. Cox regression was conducted to identify risk factors for all-cause mortality. A propensity score-matched analysis was performed at a 1:1 ratio with a caliper of 0.1 pooled standard deviations of relevant confounders.

**Results:**

The final analysis included a total of 332 patients (167 confirmed cases and 165 suspected cases), 42.77% (142/332) of the patients were 80 years of age or older and 68.67% (228/332) of them were men, 158 patients were treated with azvudine. In the matched cohort, the total mortality was 30.30% (60/198), 40 (20.20%, 40/198) patients received noninvasive ventilation and 22 (11.11%, 22/198) received invasive ventilation, 34 (17.17%, 34/198) patients were admitted to intensive care unit (ICU). The rate of shock, multiple organ damages and arrhythmia were 11.62% (23/198), 20.20% (40/198), and 12.12% (24/198), respectively. There was no significant difference on these clinical outcomes in patients treated with azvudine or not. Azvudine reduced early mortality (within 14 days from admission) (hazard ratio: 0.37, 95% confidence interval: 0.18–0.77) even after adjusting for other treatments including glucocorticoids, immunoglobin and anticoagulant therapy, but not the final in-hospital mortality of patients.

**Conclusions:**

Patients with COVID-19-related myocardial injury had a high mortality of about 30.30% (60/198). Azvudine improved the early survival of the patients but not final mortality.

## INTRODUCTION

Since the outbreak of coronavirus disease 2019 (COVID-19) in 2019, five major variants of severe acute respiratory syndrome coronavirus 2 (SARS-CoV-2) have emerged: alpha (B.1.1.7), beta (B.1.351), gamma (P.1), delta (B.1.617.2), and omicron (B.1.1.529)^[1,2]^. In comparison to early wild-type strain, the late variants are generally more transmissible and tend to produce immune escape^[1]^. Omicron (B.1.1.529) was named as a variant of concern (VOC) on November 26, 2021 by World Health Organization (WHO) and is now the dominant strain worldwide^[3]^. In comparison to prior SARS-CoV-2 variants, omicron is highly transmissible and prone to immune escape^[4]^. According to WHO, over 661 million confirmed cases of COVID-19 and over 6.7 million deaths have been reported globally as of January 13, 2023. During the omicron pandemic from December 5, 2022, to January 1, 2023, over 14.5 million cases and over 46,000 new fatalities were reported globally, representing an increase of 25% and 21% compared to the previous 28 days.

The lower respiratory tract seems to be less affected by the omicron variants, but details in difference to prior variants are not clear^[5]^. For extrapulmonary presentations, myocardial injuries in patients with COVID-19 could manifest as dysrhythmias, acute coronary syndrome (ACS), heart failure, and myocarditis^[6]^. Cardiovascular complications have been reported in 23% of the patients infected by prior SARS-CoV-2 variants^[7]^. It was reported that myocarditis accounted for death in 7% of the patients^[8]^. Left heart failure was associated with increased mortality and was present in 52% of nonsurvivors^[8]^. Importantly, cardiac involvement in COVID-19 patients might have been underestimated. For example, 78% of patients with mild-to-moderate COVID-19 who underwent cardiac magnetic resonance imaging showed abnormal findings, mostly cardiac inflammation^[9]^. In comparison to the delta variant, patients infected with the omicron variants tend to have less severe symptoms, lower rate of hospitalization and mortality^[10–12]^. Nevertheless, myocarditis has been reported in patients infected with the omicron variants^[13]^.

Treatment strategy for myocardial injuries in COVID-19 patients has been poorly developed. Antiviral agents are intuitively important from an etiological viewpoint. Molnupiravir and Paxlovid (nirmatrelvir/ritonavir) are approved for use in COVID-19 patients. The rate of hospitalization and mortality could be decreased by 30% with molnupiravir and by 89% with Paxlovid^[14]^. However, the US Food Drug Administration (FDA) approved molnupiravir only in adults and Paxlovid in persons aged ≥12 years weighing ≥40 kg with mild-to-moderate COVID-19 who are at risk for progression^[15]^. More importantly, worldwide use of molnupiravir and Paxlovid is limited due to cost issue. Azvudine is a nucleoside analog originally developed for human immunodeficiency virus (HIV) infection^[16]^. In a small randomized trial using standard antiviral treatment (interferon alpha, kaletra, and ribavirin) as control, azvudine showed promising safety and efficacy in patients with mild and common COVID-19^[17]^. Azvudine has been approved in China for use in COVID-19 patients, but the potential effects in patients with COVID-19-associated myocardial injury remain unclear.

We conducted a retrospective analysis and propensity score-matched analysis to compare all-cause mortality in COVID-19 patients with myocardial injuries receiving versus not receiving azvudine.

## METHODS

### Study design and participants

This retrospective analysis included consecutive symptomatic patients of COVID-19 with cardiac injury receiving treatment at Wuhan Union Hospital during a period from December 7, 2022, to December 30, 2022. The study was conducted in accordance with the *Declaration of Helsinki* (as revised in 2013) and approved by the ethics board of the Union Hospital, Tongji Medical College, Huazhong University of Science and Technology, Wuhan, China (NO. [2020]0073 in January 28, 2020). Due to the retrospective nature of the study, informed consent was waived.

Diagnosis of confirmed and suspected COVID-19 was established according to “Diagnosis and Treatment Program for Novel Coronavirus Pneumonia”(ninth edition)^[18]^. Suspected COVID-19 cases were defined as any one of following epidemiology histories plus two of the following clinical manifestations, or all three clinical manifestations with no regards to the epidemiology histories. Epidemiology histories included: (1) having a history of travel or residence in communities with cases reported within 14 days before the patient’s onset; (2) having a contact history with patients (a positive result of nucleic acid test of COVID-19) with 14 days before the patient’s onset; (3) having a contact history with patients with fever or respiratory symptoms from communities with cases reported 14 days before the patient’s onset; and (4) clustering occurrence of cases. Clinical manifestations included: (1) fever and/or respiratory symptoms; (2) having the imaging features of pneumonia caused by COVID-19; and (3) in the early stage, a normal or decreased total white blood cell count and a decreased lymphocyte count can be found. Definitive COVID-19 diagnosis was based on nucleic acid testing for COVID-19 or positive immunoglobulin G (IgG) and IgM antibodies targeting COVID-19. Cardiac injury was defined as the serum levels of troponin I increased above the 99th percentile upper reference limit^[19]^. Severity of the disease was classified according to “Diagnosis and Treatment Program for Novel Coronavirus Pneumonia” (ninth edition) for the treatment of adult patients with common COVID-19. All cases admitted were at least ordinary cases, that is, each of them had symptoms like fever or respiratory tract symptoms and pneumonia manifestations proved by imaging. Severe disease was defined as: (1) respiratory distress, respiratory rate ≥30 breaths/min; (2) oxygen saturation at <93% at rest state without oxygen therapy; (3) arterial partial pressure of oxygen (PaO_2_)/oxygen concentration (FiO_2_) at ≤300 mmHg (1 mmHg = 0.133 kPa), for high altitude areas (above 1 kilometer), PaO_2_/FiO_2_ values should be adjusted based on equation of PaO_2_/FiO_2_ × (atmospheric pressure [mmHg]/760), and (4) >50% lesions progression within 24 to 48 hours in pulmonary imaging. Critical disease was defined as having at least one of the following conditions: (1) requirement of mechanical ventilation; (2) shock; and (3) requirement of intensive care unit (ICU) admission.

### Data collection

Medical records were reviewed by a research team in Clinic Center of Human Gene Research, Union Hospital. Demographic characteristics (age and sex), comorbidities, symptoms, signs, laboratory findings, treatments and clinical outcomes were extracted and cross-checked. Patients with missing key data were excluded from the analysis. Azvudine was an antiviral drug used in the initial cohort. In our data, there are two distinct criteria on azvudine use: (1) use within 7 days from admission and (2) use as an initial therapy at baseline. The outcome of primary interest was all-cause mortality. Other outcomes included the rate of shock, multiple organ damage, arrhythmia, ICU admission and advanced oxygen support (transnasal high-flow oxygen inhalation, noninvasive ventilation and invasive ventilation).

### Statistical analysis

Baseline characteristics were presented as mean (standard deviation [SD]) or median (interquartile range [IQR]) for continuous variables and percentages for categorical variables, respectively. Propensity score matching was conducted at a 1:1 ratio with a caliper of 0.1 pooled SDs based on the following variables: demographics, signs and symptoms at admission, laboratory examination, and comorbidities. Absolute standardized differences <25%, the threshold suggested by Robin and Stuart^[20]^, are considered inconsequential. To determine whether the results of our analysis was confounded by biases associated with prevalent drug use, we conducted covariate adjustment analyses using prevalent drugs (i.e., glucocorticoid, anticoagulant drug and intravenous immunoglobulin).

Pearson’s *χ^2^* test and McNemar’s test for categorical variables and Wilcoxon rank-sum test and Student’s *t* test for paired samples for continuous variables were used to between-group comparisons, respectively. Cox proportional hazard regression was used to assess the potential association of azvudine use with outcomes. The cumulative probabilities of survival in the two treatment groups were estimated according to the Kaplan–Meier method. Results are presented as hazard ratio and its 95% confidence interval (95% CI). Regression analysis of all-cause mortality was adjusted by glucocorticoid, anticoagulant drug, and intravenous immunoglobulin. Subgroup analyses were used to evaluate the homogeneity of the association of azvudine with all-cause mortality. A sensitivity analysis was conducted using inverse probability of treatment weighting (IPTW). All statistical analyses were carried out using SAS version 9.3 (SAS Institute, Inc., Cary, NC, USA). A 2-sided *P* value <0.05 was considered statistically significant.

## RESULTS

### Baseline characteristics

**Figure 1.**
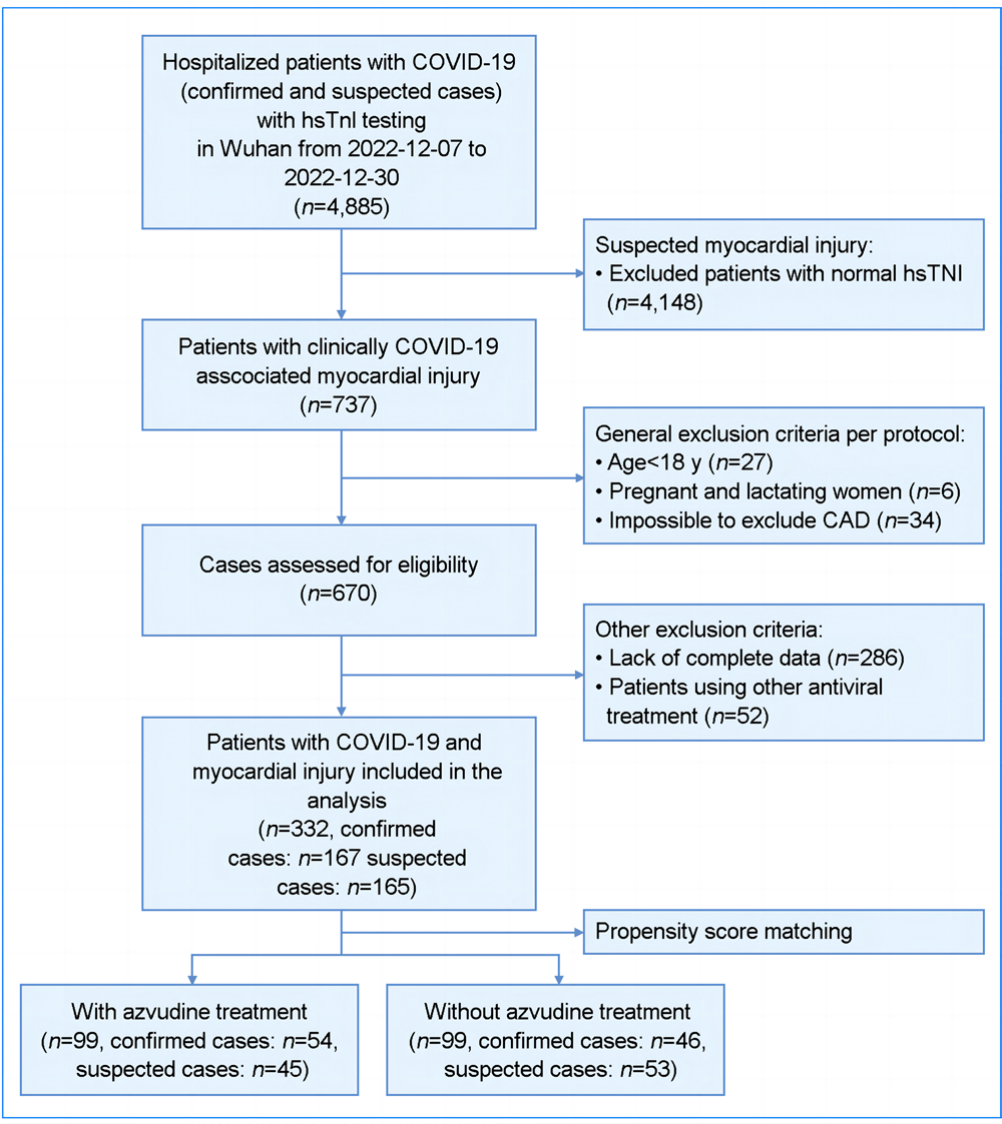
**Flow chart of the study cohort**. CAD: cardiovascular disease; COVID-19: coronavirus disease 2019; hsTnI: high sensitivity troponin I.

The final analysis included a total of 332 patients: 165 (49.70%) suspected cases and 167 (50.30%) confirmed cases. The propensity score-matched analysis included 198 cases (100 confirmed cases and 98 suspected cases) (Figure 1). Demographic and clinical characteristics before and after propensity score matching are shown in Table 1. Details of the matching are shown in Supplementary Figure 1, 57.23% (190/332) of the patients were 80 years of age or younger and 68.67% (228/332) were male. Fever was the most common symptom in both groups. 63.25% (210/332) of the patients had hypertension and 37.95% (126/332) had co-existing cardiovascular diseases. Common laboratory findings included elevated neutrophil count, C-reactive protein (CRP) and D-dimer. The male sex was over-presented in the azvudine group.

**Table 1:** Demographic and clinical characteristics at the baseline, by the use of azvudine in patients with myocardial injuries, before and after propensity score matching

	Before propensity-matching				After propensity-matching			
	Total	Without azvudine use (*n* = 174)	With azvudine use (*n* = 158)	*P* values*	Total	Without azvudine use (*n* = 99)	With azvudine use (*n* = 99)	*P* values*
Classification, *n* (%)								
Confirmed cases	167 (50.30)	97 (55.75)	70 (44.30)	0.079	100 (50.51)	54 (54.55)	46 (46.46)	0.418
Suspected cases	165 (49.70)	77 (44.25)	88 (55.70)	0.079	98 (49.49)	45 (45.45)	53 (53.54)	0.418
Age, *n* (%)								
≤80 y	190 (57.23)	101 (58.05)	89 (56.33)	0.752	120 (60.61)	60 (60.61)	60 (60.61)	1.000
>80 y	142 (42.77)	73 (41.95)	69 (43.67)	0.752	78 (39.39)	39 (39.39)	39 (39.39)	1.000
Sex, *n* (%)								
Female	104 (31.33)	64 (36.78)	40 (25.32)	0.025	58 (29.29)	29 (29.29)	29 (29.29)	1.000
Male	228 (68.67)	110 (63.22)	118 (74.68)	0.025	140 (70.71)	70 (70.71)	70 (70.71)	1.000
Comorbidity, *n* (%)								
Hypertension	210 (63.25)	113 (64.94)	97 (61.39)	0.503	127 (64.14)	61 (61.62)	66 (66.67)	0.457
Diabetes	89 (26.81)	53 (30.46)	36 (22.78)	0.115	57 (28.79)	31 (31.31)	26 (26.26)	0.425
Cardiovascular disease	126 (37.95)	62 (35.63)	64 (40.51)	0.361	71 (35.86)	34 (34.34)	37 (37.37)	0.662
Cerebrovascular disease	70 (21.08)	40 (22.99)	30 (18.99)	0.372	41 (20.71)	20 (20.20)	21 (21.21)	0.858
Chronic pulmonary disease	61 (18.37)	38 (21.84)	23 (14.56)	0.087	28 (14.14)	13 (13.13)	15 (15.15)	0.683
Chronic renal disease	62 (18.67)	41 (23.56)	21 (13.29)	0.017	29 (14.65)	14 (14.14)	15 (15.15)	0.828
Cancer	35 (10.54)	21 (12.07)	14 (8.86)	0.342	21 (10.61)	12 (12.12)	9 (9.09)	0.493
Symptom								
Onset of symptom to hospital admission, days (mean ± SD)	8.29 ± 6.46	8.61 ± 7.94	7.95 ± 4.28	0.490	8.13 ± 6.15	8.08 ± 7.28	8.17 ± 4.79	0.923
Fever, *n* (%)	227 (68.37)	116 (66.67)	111 (70.25)	0.483	135 (68.18)	69 (69. 70)	66 (66.67)	0.662
Other symptoms, *n* (%)	323 (97.29)	168 (96.55)	155 (98.10)	0.385	192 (96.97)	96 (96.97)	96 (96.97)	1.000
Disease severity, *n* (%)								
Moderate	27 (8.13)	18 (10.34)	9 (5.70)	0.159	18 (9.09)	9 (9.09)	9 (9.09)	0.988
Severe	202 (60.84)	108 (62.07)	94 (59.49)	0.159	115 (58.08)	57 (57.58)	58 (58.59)	0.988
Grave	103 (31.02)	48 (27.59)	55 (34.81)	0.159	65 (32.83)	33 (33.33)	32 (32.32)	0.988
Signs (mean ± SD)								
Temperature, ℃	37.33 ± 1.08	37.23 ± 1.07	37.44 ± 1.09	0.032	37.32 ± 1.09	37.32 ± 1.11	37.32 ± 1.07	0.994
Respiration rate, breaths/min	21.99 ± 13.05	22.70 ± 17.68	21.20 ± 3.68	0.846	21.52 ± 3.84	21.59 ± 3.49	21.45 ± 4.19	0.803
Heart rate, bpm	86.73 ± 18.63	87.40 ± 19.35	86.00 ± 17.83	0.846	86.73 ± 19.47	86.49 ± 19.23	86.96 ± 19.80	0.868
Systolic blood pressure, mmHg	132.08 ± 23.33	130.71 ± 24.36	133.58 ± 22.13	0.296	131.40 ± 23.25	130.71 ± 25.30	132.09 ± 21.11	0.682
Diastolic blood pressure, mmHg	76.89 ± 14.39	76.94 ± 15.48	76.82 ± 13.14	0.860	77.65 ± 14.66	77.42 ± 15.75	77.87 ± 13.55	0.835
Laboratory results (mean ± SD)								
Highest hsTnI, ng/L	2558.66 ± 16404.84	1729.98 ± 10,751.00	3471.26 ± 20,938.00	0.170	1195.31 ± 5953.12	1184.51 ± 7238.93	1206.11 ± 4340.33	0.980
White blood cell count, ×10^9^/L	7.76 ± 4.67	8.01 ± 4.97	7.48 ± 4.31	0.301	7.79 ± 4.68	7.92 ± 5.02	7.66 ± 4.37	0.700
Neutrophil count, ×10^9^/L	6.50 ± 4.53	6.70 ± 4.85	6.27 ± 4.14	0.456	6.52 ± 4.57	6.62 ± 4.95	6.42 ± 4.18	0.761
Lymphocyte, ×10^9^/L	0.75 ± 0.55	0.77 ± 0.55	0.73 ± 0.55	0.683	0.75 ± 0.58	0.77 ± 0.54	0.74 ± 0.62	0.740
Hemoglobin, g/L	117.79 ± 25.31	115.85 ± 27.43	119.92 ± 22.64	0.277	119.46 ± 24.52	120.47 ± 26.01	118.44 ± 23.03	0.566
C-reactive protein, mg/L	87.01 ± 71.52	85.99 ± 78.56	88.13 ± 63.10	0.186	92.37 ± 76.19	95.95 ± 85.96	88.79 ± 65.23	0.518
Alanine aminotransferase, U/L	38.55 ± 77.36	43.60 ± 103.39	32.98 ± 27.93	0.527	32.34 ± 22.94	31.08 ± 20.88	33.61 ± 24.86	0.451
Blood urea nitrogen, mmol/L	14.22 ± 11.6	15.67 ± 12.71	12.63 ± 10.03	0.018	13.12 ± 11.15	13.42 ± 11.90	12.82 ± 10.40	0.706
eGFR, mL/min/1.73 m^2^								
≤45	193 (58.13)	85 (48.85)	108 (69.35)	0.000	123 (62.12)	61 (61.62)	62 (62.63)	0.866
>45	139 (41.87)	89 (51.15)	50 (31.65)	0.000	75 (37.88)	38 (38.38)	37 (37.37)	0.866
D-dimer, ng/L	4.56 ± 7.38	4.89 ± 7.23	4.20 ± 7.54	0.523	4.68 ± 8.08	4.63 ± 7.42	4.73 ± 8.72	0.936

* *P* values indicate differences between patients with use of azvudine and without use of azvudine. *P* <0.05 was considered statistically significant.

eGFR: estimated glomerular filtration rate; hsTnI: high sensitivity troponin I; SD: standard deviation.

### Adverse clinical events

A total of 51 (15.36%, 51/332) patients were admitted to ICU (Table 2). Severe complications included shock in 34 (10.24%, 34/332) patients, multiple organ damages in 57 (17.17%, 57/332) patients and arrhythmia in 34 (10.24%, 34/332) patients. One patient received extracorporeal membrane oxygenation (ECMO) treatment. A total of 91 (27.41%, 91/332) patients deceased. With the exception of advanced oxygen supply, these adverse clinical events did not differ between the azvudine and nonazvudine groups. Patients treated with azvudine were less likely to receive transnasal high-flow oxygen inhalation (*P* = 0.026), but had a higher risk of receiving noninvasive ventilation (*P* = 0.003) and invasive ventilation (*P* = 0.048). However this difference was diminished after propensity-matching.

**Table 2:** Clinical outcomes by the use of azvudine in patients with myocardial injuries, before and after propensity score matching

Clinical outcomes	Before propensity score matching				After propensity score matching			
	Total	Without azvudine use (*n* = 174)	With azvudine use (*n* = 158)	*P* values*	Total	Without azvudine use (*n* = 99)	With azvudine use (*n* =99)	*P* values*
Transnasal high-flow oxygen inhalation use, *n* (%)	67 (20.18)	27 (84.48)	40 (25.32)	0.026	45 (22.73)	19 (19.19)	26 (26.26)	0.235
Noninvasive ventilation use, *n* (%)	60 (18.07)	21(12.07)	39 (24.68)	0.003	40 (20.20)	16 (16.16)	24 (24.24)	0.157
Invasive ventilation use, *n* (%)	31 (9.34)	11 (6.32)	20 (12.66)	0.048	22 (11.11)	10 (10.10)	12 (12.12)	0.651
ECMO use, *n* (%)	1 (0.30)	1 (0.57)	0 (0.00)	0.340	1 (0.51)	1 (1.01)	0 (0.00)	0.316
ICU admission, *n* (%)	51 (15.36)	23 (13.22)	28 (17.72)	0.256	34 (17.17)	17 (17.17)	17 (17.17)	1.000
Multiple organ dysfunction, *n* (%)	57 (17.17)	31 (17.82)	26 (16.46)	0.743	40 (20.20)	22 (22.22)	18 (18.18)	0.479
Cardiac arrhythmia, *n* (%)	34 (10.24)	20 (11.49)	14 (8.86)	0.429	24 (12.12)	14 (14.14)	10 (10.10)	0.384
Shock, *n* (%)	34 (10.24)	21 (12.07)	13 (8.23)	0.249	23 (11.62)	16 (16.16)	7 (7.07)	0.046
Death, *n* (%)	91 (27.41)	45 (25.86)	46 (29.11)	0.507	60 (30.30)	31 (31.31)	29 (29.29)	0.757

* *P* values indicate differences between patients with use of azvudine and without use of azvudine. *P* <0.05 was considered statistically significant.

ECMO: extracorporeal membrane oxygenation; ICU: intensive care unit.

### All-cause mortality of patients

**Figure 2.**
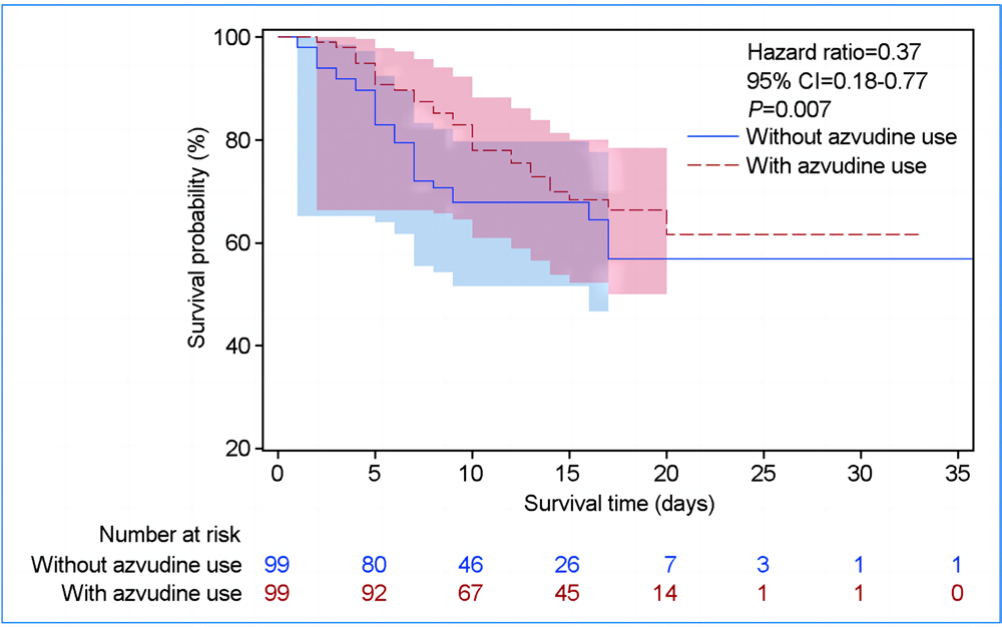
**Kaplan–Meier plot for all-cause mortality by the use of azvudine in patients with myocardial injuries**. 95% CI: 95% confidence interval.

Compared with pre-match patients, those in the 1:1 propensity-matched cohort showed substantially improved balance (in terms of reduced absolute standardized differences <25%) across the 29 baseline characteristics (Supplementary Figure 1). After propensity-matching, all-cause mortality was 30.30%, with no difference between azvudine (29.29%) and nonazvudine group (31.31%). Azvudine significantly reduced early mortality (within 14 days of admission) (hazard ratio [HR]: 0.37, 95% CI: 0.18–0.77, *P* = 0.007) (Table 3) but not final mortality (Figure 2). Such effect remained even after adjusting for other treatments, including glucocorticoid, anticoagulant drugs, and intravenous immunoglobin (Table 3).

**Table 3:** Hazard ratios of azvudine use on COVID-19 death in patients with myocardial injuries adjusted by other medical treatments after propensity score matching

Adjustment	Hazard ratio (95% CI)	*P* value
Glucocorticoid	0.31 (0.13–0.71)	0.006
Anticoagulants	0.43 (0.20–0.92)	0.030
Glucocorticoid and anticoagulants	0.35 (0.14–0.84)	0.020
Intravenous immunoglobulin	0.36 (0.16–0.80)	0.011
Glucocorticoid and intravenous immunoglobulin	0.32 (0.13–0.75)	0.009
Anticoagulants and intravenous immunoglobulin	0.43 (0.19–0.98)	0.045
Glucocorticoid, anticoagulants, and intravenous immunoglobulin	0.37 (0.15–0.92)	0.033

95% CI: 95% confidence interval.

**Figure 3.**
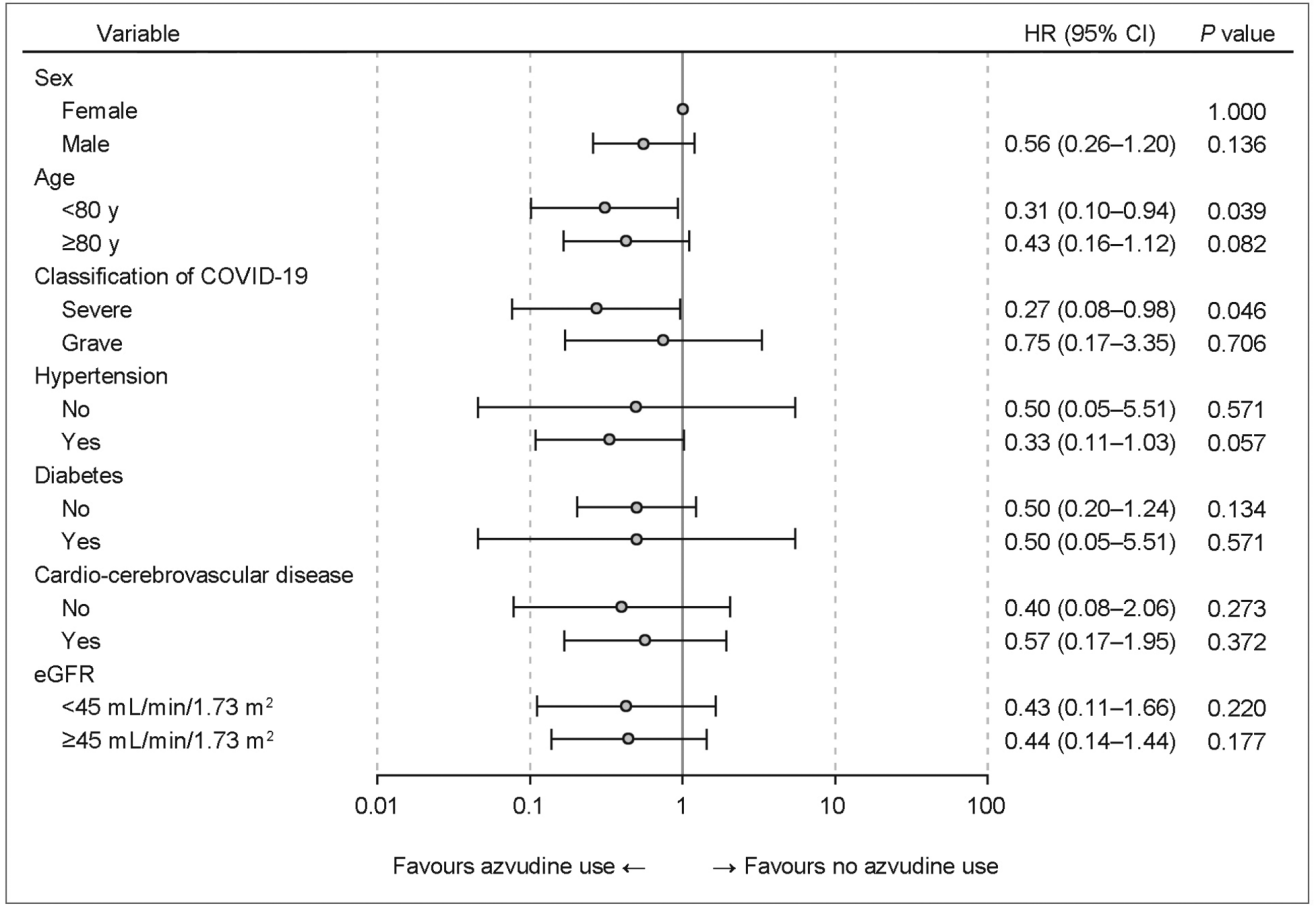
**Association of azvudine with COVID-19 death in subgroup analysis of the propensity score-matched cohort**. 95% CI: 95% confidence interval; COVID-19: coronavirus disease 2019; eGFR: estimated glomerular filtration rate; HR: hazard ratio.

Azvudine treatment was associated with lower mortality in the subgroup analysis that only included patients with severe disease (HR: 0.27, 95% CI: 0.08–0.98), but not with critical disease and in patients <80 years of age (HR: 0.31, 95% CI: 0.10–0.94) (Figure 3). No difference was observed in subgroup analysis based on sex, renal function and comorbidities including hypertension, diabetes, and cardiocerebral vascular disease.

As listed in Supplementary Table 1, the estimated effect size of azvudine was close to the value estimated after propensity-matching, and remained statistically significant after IPTW (HR: 0.67, 95% CI: 0.47–0.96; *P* = 0.029) and adjustment for propensity scores (HR: 0.35, 95% CI: 0.15–0.79). It suggested the robustness of our analysis.

## DISCUSSION

Angiotensin-converting enzyme 2 (ACE2), the SARS-CoV-2 cell entry receptor, is expressed in cardiac muscle cells and endothelial cells^[21]^. Viral invasion into cardiac cells results in inflammation and production of free radicals, relative hypoxia, and microvascular thrombosis, thus causing direct myocardial cell damage^[21,22]^. On the other hand, ACE2 is a dominant regulator of the renin–angiotensin system (RAS). SARS-CoV-2 downregulates ACE2 and subsequently hyperactivates Ang II/AT1 system, leading to vasoconstrictive, proinflammatory, and prooxidant effects^[23]^. During SARS-CoV-2 infection the activated immune system generates cytokine storm that could promote cardiomyocyte apoptosis and fibrosis^[24]^, inflammatory cell infiltration, and expression of procoagulant factors, contributing to propagation of microcirculatory lesions and endothelial dysfunction^[25]^. Elevated circulatory macrophages might interact with preexisting atherosclerotic plaques possibly causing myocardial infarction^[26]^. As a result, myocardial injury including myocarditis, heart failure, and myocardial infarction may manifest through one or more of these underlying mechanisms. Whether different variants of SARS-COV-2 infection affect the cardiovascular system in their unique way is unknown and more studies are required to further delineate the potential mechanisms.

Currently, there is no specific therapy for COVID-19-induced myocardial injury. Several cases published in literature reported treatment strategies including antiviral drugs, corticosteroid, immunoglobulin, tocilizumab, and antiplatelets, especially for patients with ACS^[27,28]^. Administration of nutritional supplements including coenzyme Q10, creatine phosphate, vitamin C, and deep sea fish oil was suggested in patients with COVID-19^[29]^ and myocardial injury. None of the completed and on-going trials of immunosuppressants in COVID-19 patients is designed for myocarditis^[27]^.

Azvudine is the first double‐target nucleoside drug with a broad spectrum of antiviral effects including HIV, hepatitis C virus and hepatitis B virus, etc^[17]^. In 2020, azvudine was first proved to shorten the mean time of the first nucleic acid negative conversion in mild and common COVID‐19 patients^[17]^. On July 25, 2022, the National Medical Products Administration granted conditional authorization for azvudine to treat COVID-19^[30]^. In this study, patients with myocardial injury had a high mortality of about 30.30%, indicating the severe nature of such cases. Furthermore, only patients with moderate (8.13%)/severe (60.84%)/critical (31.02%) disease were included. Limited preparedness to face the challenge of large number of infected patients within a very short period of time might have also contributed to the poor outcome.

In the current study, the use of azvudine was associated with significantly prolonged survival time but not with the overall mortality. Results of the subgroup analysis suggested that younger patients (<80 years) with severe disease could benefit from azvudine use. Considering the multisystem involvement of the disease and the complexity of mechanisms through which viral infection causes myocardial injury, antiviral medication is necessary, but only one part of the overall treatments. Improved short-term survival but not overall mortality in the azvudine group is consistent with this view.

The current study has several limitations. First, this is a single-centered retrospective study with limited sample size. Second, patients with mild disease were not included in the analysis. Third, RNA testing results were not available in a significant proportion of the patients.

## CONCLUSIONS

COVID-19 patients with myocardial injury have a high risk of death and poor outcome. The use of azvudine was associated with improved 14-day mortality but not the overall mortality during the study period.

## FUNDING

This study was supported by the National Natural Science Foundation of China (No: 81830014 and 91949201).

## AUTHOR CONTRIBUTIONS

RC and YG drafted the manuscript. KH, SD, and LW participated in the research design and the performance of the research. YG, JW, MG, and HLH participated in data collection. HWJ analyzed the data and participated in the research design. KH and HWJ revised the manuscript.

## CONFLICT OF INTEREST STATEMENT

The authors declare that they have no conflict of interest with regard to the content of this manuscript.

## DATA SHARING STATEMENT

The datasets analyzed during the current study are available from the corresponding author on request.

## Supplementary Material
