# Supplementary material for: All-cause mortality in moderate and severe COVID-19 patients with myocardial injury receiving versus not receiving azvudine: a propensity score-matched analysis
Source: Cardiol Plus. 2023 May 31;8(2):103–10. doi: 10.1097/CP9.0000000000000049 (PMC10364645; doi:10.1097/CP9.0000000000000049)
Supplement: Supplementary file 1 [file cp9-8-103-s001.pdf]

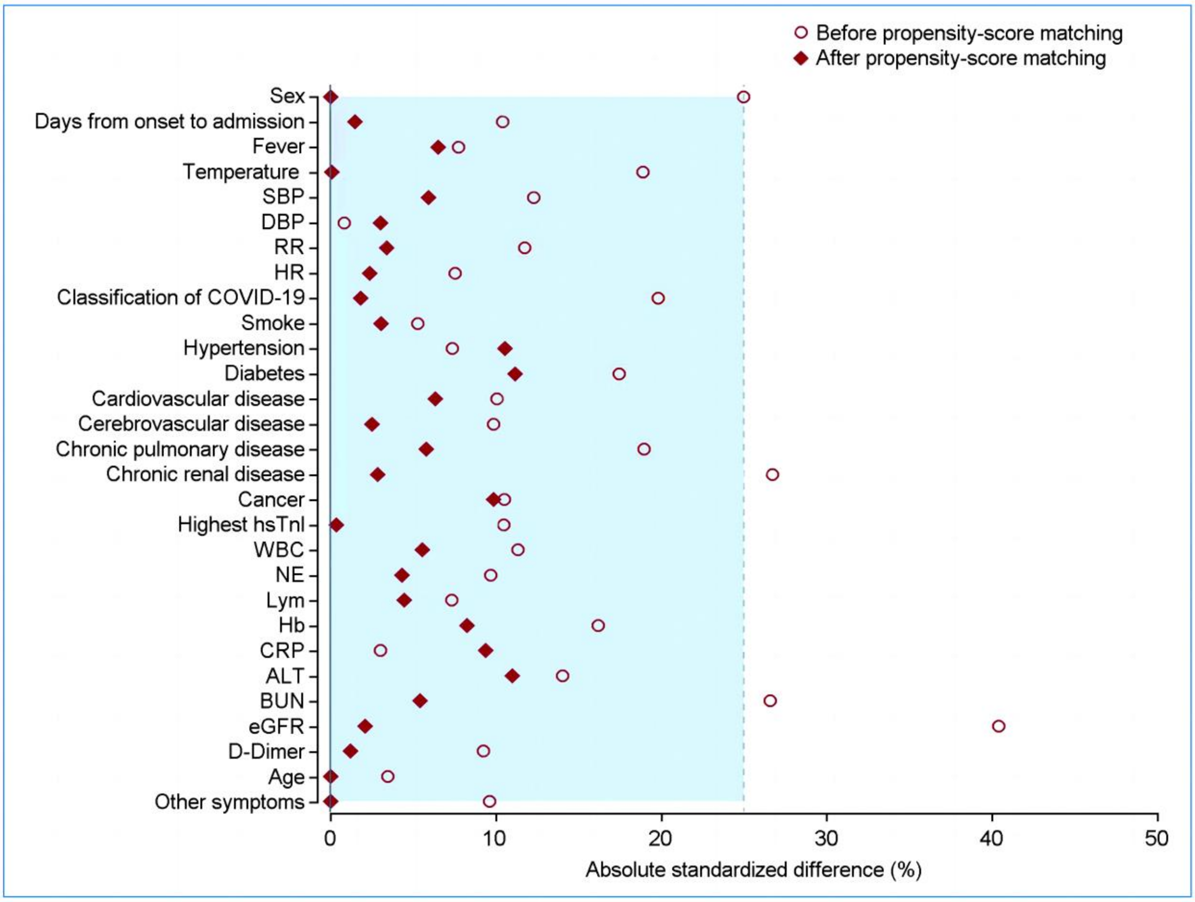

Figure S1. Love plot displaying standardized differences for the baseline characteristics by the use of azvudine in patients with myocardial injuries, before and after propensity score matching.

SBP: systolic blood pressure; DBP: diastolic blood pressure; RR: respiratory rate; HR: heart rate; COVID-19: coronavirus disease 2019; hsTnI: high sensitivity troponin I; WBC: white blood cell; NE: neutrophil; Lym: Lymphocyte; Hb: hemoglobin; CRP: C-reactive protein; ALT: alanine aminotransferase; BUN: blood urea nitrogen; eGFR: estimated glomerular filtration rate; DD: D-dimer.
