# Supplementary material for: All-cause mortality in moderate and severe COVID-19 patients with myocardial injury receiving versus not receiving azvudine: a propensity score-matched analysis
Source: Cardiol Plus. 2023 May 31;8(2):103–10. doi: 10.1097/CP9.0000000000000049 (PMC10364645; doi:10.1097/CP9.0000000000000049)
Supplement: Supplementary file 2 [file cp9-8-103-s002.pdf]

Table S1 Sensitivity analysis using inverse probability of treatment weighting (IPTW) and covariate adjustment based on propensity score for hazard ratio of azvudine use at baseline on COVID-19 death in a propensity score matched cohort.

**Method**	**Hazard ratio (95% CI)**	***P* value**
IPTW	0.672 (0.470-0.959)	0.029
Covariate adjustment	0.345 (0.151-0.791)	0.012

95% CI: 95% confidence interval; IPTW: inverse probability of treatment weighting.
